# 

**Published:** 1887-04

**Authors:** 


					﻿-AT=>KIL, ISQT’-
SERIES
1855
NEW SERiE^
Vol. IV.-No. 2./


rfDUBNAL


sTyroorc J
W.F.WESTMORErXND.|jH
\ZM.M1LLER. M.D.LL .D.JC
JAMES ACRAY. MJ}. 11
*i> '■>- --- fc<
dBY JAMES P.HARRISON&CgS
Atlanta .GA.
SUBSCRIPTION: S2.SO A YEAR.
				

